# Intravenous thrombolysis in the emergency department for the treatment of acute ischaemic stroke

**DOI:** 10.1136/emj.2007.053033

**Published:** 2008-06-24

**Authors:** A Semplicini, V Benetton, L Macchini, A Realdi, R Manara, C Carollo, E Parotto, V Mascagna, M Leoni, L A Calò, A C Pessina, F Tosato

**Affiliations:** 1 Department of Clinical and Experimental Medicine, University of Padua Medical School and Azienda Ospedaliera, Padua, Italy; 2 Department of Neurological Sciences, University of Padua Medical School and Azienda Ospedaliera, Padua, Italy; 3 Emergency Department, University of Padua Medical School and Azienda Ospedaliera, Padua, Italy

## Abstract

**Background and aims:**

Thrombolytic therapy with intravenous recombinant tissue plasminogen activator (rt-PA) improves outcome in patients with ischaemic stroke treated within 3 h of symptom onset, but its extended implementation is limited. A pilot study was designed to verify whether evaluation of patients with acute ischaemic stroke and their treatment with intravenous rt-PA in the emergency department (ED), followed by transportation to a semi-intensive stroke care unit, offers a safe and effective organisational solution to provide intravenous thrombolysis to acute stroke patients when a stroke unit (SU) is not available.

**Methods:**

After checking for inclusion and exclusion criteria, ED doctors contacted the stroke team with a single page, located family members and urgently obtained computed tomography scan and laboratory tests. A stroke team investigator clinically assessed the patient, obtained written informed consent and supervised intravenous rt-PA in the ED. After treatment, the patient was transferred to the SU for rehabilitation and treatment of complications, under supervision of the same stroke team investigator.

**Results:**

52 patients were treated with intravenous rt-PA within 3 h of symptom onset. 20 patients (38%) improved neurologically after 24 h, the number increased to 30 (58%) after one week. At 3 months 22 patients had a favourable outcome (43%). The 3-month mortality rate was 12%. Symptomatic cerebral haemorrhage was observed in two patients (4%).

**Conclusions:**

Intravenous rt-PA administration in the ED is an effective organisational solution for acute ischaemic stroke when an SU is not established.

Thrombolytic therapy with intravenous recombinant tissue plasminogen activator (rt-PA) has been shown to improve outcome in patients with ischaemic stroke treated within 3 h of symptom onset.^1^ In the United States, however, 69% of hospitals do not use thrombolysis at all and only 1% of stroke patients receive thrombolytics, mostly at hospitals with a high volume of stroke patients.^2^ This implies that only immediate referral of stroke patients to strategically placed, certified, high-volume stroke centres with continuously available, dedicated and sophisticated stroke teams, analogous to the manner in which trauma patients are routed to certified trauma centres, may increase the thrombolysis rate.^2^

Until recently, the extended use of thrombolytic therapy with intravenous rt-PA has been prevented in Europe by concerns about its safety profile, as it increases the risk of cerebral haemorrhage, and its efficacy in clinical practice where stroke units (SU) are not available. The SITS–MOST trial, aiming to define the risks of rt-PA administration to patients with acute ischaemic stroke within 3 h of symptom onset in experienced stroke centres in Europe, has shown a favourable risk–benefit ratio.^3^ It is still debated how to implement thrombolytic therapy, once its extended use has been approved. In fact, widespread experience is limited and the number of SU in most countries is small and unevenly distributed, so that transportation of the patient to the closest SU may delay treatment and may further reduce the number of candidate patients.

In 2000, an SU was not established in our institution and intravenous rt-PA was not registered for the treatment of stroke in Italy. Therefore, we designed a pilot clinical study to investigate how to implement intravenous thrombolysis for the treatment of ischaemic stroke. The study assessed the safety and outcome of intravenous thrombolysis with rt-PA in the emergency department (ED), followed by transfer of patients to a semi-intensive SU for follow-up monitoring, further treatment and rehabilitation.

## METHODS

Stroke team collaboration was established at the University Hospital of Padua, a large academic city hospital with more than 100 000 consultations per year in the ED. The aim of stroke team collaboration was to increase awareness about appropriate treatments of acute ischaemic stroke (ie, intravenous and intra-arterial thrombolysis, as well as endovascular procedures). In particular, its aim was to evaluate how to implement off-label intravenous thrombolysis with rt-PA in acute ischaemic stroke, before the institution of the SU and the formal approval of rt-PA in stroke patients by the European and local regulatory agencies. This pilot clinical trial was approved by the local Ethical Committee in 1999 and lasted from 1 January 2000 to 30 June 2007.

The stroke team was composed of internists, neurologists, neuroradiologists, emergency doctors and anaesthetists. Its first task was to train the doctors of the ED about triage and the management of patients with acute stroke, so that any patient admitted to the ED with suspected ischaemic stroke was immediately triaged by ED doctors for indications for systemic thrombolysis. The inclusion/exclusion criteria for thrombolysis were those of the National Institute of Neurological Disorders and Stroke (NINDS) trial.^1^ The most common reasons against rt-PA treatment were age greater than 80 years, time window greater than 3 h, unclear onset of symptoms, rapidly improving symptoms, mild stroke (National Institutes of Health Stroke Scale (NIHSS) score ⩽4), severe stroke (NIHSS score ⩾25) and pretreatment with anticoagulants. Pretreatment with aspirin or other antiplatelet agents was not an exclusion criterion. Special attention was paid to exclude patients with uncontrolled hypertension, but in a few patients blood pressure control was obtained with one or two small intravenous labetalol bolus injection(s) before thrombolysis.^1^

If the patient fulfilled the inclusion/exclusion criteria, ED doctors contacted the stroke team on a convenience basis, mostly between 08:00 and 20:00 hours during weekdays, with a single page, located family members, urgently obtained a computed tomography (CT) scan and laboratory tests. One stroke team investigator, previously authorised by the local Ethical Committee, convened for clinical assessment, to verify inclusion/exclusion criteria and to obtain a written informed consent from the patient or a close relative, while the CT scan was performed. Neurological deficit on admission was measured by the NIHSS.

rt-PA (Actilyse, Boehringer Ingelheim, Germany) was administered intravenously at a dose of 0.9 mg/kg body weight (maximum dose 90 mg), with 10% given as a bolus and the remaining 90% as a constant infusion over one hour under direct stroke team supervision in the ED. The patients had to be treated within 3 h from symptom onset.

After thrombolytic infusion, the patients were transferred to a semi-intensive SU, under supervision of the same stroke team investigator, to be followed up for the treatment of complications and rehabilitation. Clinical monitoring was performed every 2 h for the first 24 h. Blood pressure, heart rate and, when necessary, oxygen saturation were non-invasively monitored up to 72 h after stroke or longer, if necessary.

A follow-up CT scan was performed 24 h after thrombolysis in all patients and whenever the neurological condition worsened. Further CT or magnetic resonance imaging (MRI) studies were left to the discretion of the treating doctor. Neither heparin, aspirin nor other antiplatelet agents were given for 24 h after thrombolysis. Antihypertensive therapy was given according to the American Heart Association guidelines^4^ and our previous report.^5^ Rehabilitation therapy was started within 48 h after stroke onset.

Early neurological improvement was defined as a decrease of 4 or more points in the NIHSS score from baseline, or complete resolution of the neurological deficit after 24 h. Early neurological deterioration was defined as an increase of 4 or more points in the NIHSS score in the same time interval.

Telephone or clinic follow-up data were obtained from the patients and/or caregiver 3 months after treatment. Outcome was assessed by modified Rankin scale (mRS), 3-month fatality rate and symptomatic (fatal and non-fatal) intracranial haemorrhage (SICH). A good outcome was defined as an mRS score from 0 to 2, a poor outcome as death or an mRS score from 3 to 5 (death or dependence).

Cerebral bleedings were categorised according to the criteria published by Fiorelli *et al*^6^ for the European Cooperative Acute Stroke Study I (ECASS I) cohort: haemorrhagic infarctions with small petechial haematoma (HI1); haemorrhagic infarctions with more confluent petechiae (HI2); parenchymal haematoma less than 30% of the infarcted area with some mild space-occupying effect (PH1); parenchymal haematoma greater than 30% of the infarcted area with significant space-occupying effect or clot remote from the infarcted area (PH2). SICH was defined as any CT/MRI-documented haemorrhage, either within the infarcted area or in a remote area, concomitant with an increase of 4 or more points in the NIHSS score. Fatal SICH was defined as a CT/MRI-detected haemorrhage associated with in-hospital death.

Descriptive statistical analysis was performed with SPSS version 13.0. Continuous variables are expressed as mean ± SD and as median with range for variables with a non-normal distribution, whereas categorical variables are expressed as counts and percentages, to make comparisons with the NINDS results.^1^

## RESULTS

During the study period (1 January 2000 to 30 June 2007), all ED consultations for focal neurological deficits were monitored during five randomly selected weeks. The median number of hospital admissions per week during the monitoring period was 16 (six for acute ischaemic stroke, four for intracerebral haemorrhage, two for transient ischaemic attack and four for other diseases). The median “symptom to door” time was 45 minutes (range 15 minutes to 6 h) and the rate of intravenous rt-PA treatment in acute ischaemic stroke was 7%.

As patient selection and supervision could be done only by stroke team investigators authorised by the Ethical Committee, the study cohort was enrolled on a convenience basis and 52 patients with ischaemic stroke were treated with intravenous rt-PA. Their clinical characteristics are reported in table 1. They started treatment within 3 h of symptom onset. In this cohort, the mean delay between symptom onset and hospital admission was 39 minutes, from admission to CT 48 minutes and the median door-to-needle time (delay from admission to rt-PA treatment) 58 minutes.

**Table 1:** Clinical characteristics of patients enrolled in the present study, in comparison with the NINDS study^1^

	Present study	NINDS study
Number (% female)	52 (42)	312 (42)
Age, years (SD)	66 (12)	67 (10)
NIHSS score (range)	13 (5–23)	14 (1–37)
Stroke subtypes (%)		
Small-vessel occlusive	11	19
Cardioembolic	39	35
Large-vessel occlusive	27	42
Other	23	3
SBP, mm Hg (SD)	147 (22)	155 (22)
DBP, mm Hg (SD)	77 (13)	85 (12)
Glucose, mg/dl (SD)	130 (59)	149 (76)
Mortality within 7 days (%)	3 (6)	16 (5)
Symptomatic haemorrhage (%)	2 (4)	20 (6,4)

DBP, diastolic blood pressure; NIHSS, National Institutes of Health Stroke Scale; NINDS, National Institute of Neurological Disorders and Stroke; SBP, systolic blood pressure.

Thirty-four patients (65%) were admitted with a moderate (NIHSS score <15) and 18 (35%) with a serious stroke (NIHSS score 15–23). According to the Oxford Community Stroke Project classification, 15 (29%) were total anterior circulation infarct, 28 (54%) were partial anterior circulation infarct, six (11%) were lacunar infarction and three (6%) were posterior circulation infarct. According to the TOAST classification, the pathogenesis was cardioembolic in 20 (39%), atherosclerotic in 14 (27%), lacunar in six (11%) and from other or unidentified causes in 12 (23%).

After 24 h, 20 patients (38%) improved, 27 patients (52%) were in a steady condition and five patients (10%) got worse (table 2). After one week, 30 patients (58%) improved neurologically, 14 patients (27%) were in a steady condition and eight patients (15%) got worse or died. At 3 months, 22 patients (43%) had an mRS of 2 or less (0–2), 29 patients (57%) had an mRS of 3 or greater (3–5) or had died and one patient was lost to follow-up.

**Table 2:** Improvement by NIHSS scores 24 h after stroke onset, by the time to treatment: comparison between the present study and NINDS study^1^

Time to treatment after stroke onset (minutes)	Present study		NINDS study	
	Patients with improvement/treated	%	Patients with improvement/treated	%
0–90	0/0	0	87/157	55
90–180	20/52	38	60/155	39
0–180	20/52	38	147/312	47

NINDS, National Institute of Neurological Disorders and Stroke.

No complications were observed during intravenous infusion in the ED, except for minor gingival bleeding in a few patients. The 3-month mortality rate was 12%: three patients died within the first week (one from myocardial infarction and two from respiratory failure) and another three within 3 months (one from cerebral haemorrhage 7 days after thrombolysis, one from pulmonary embolism and one from candida sepsis).

Control CT/MRI scan revealed cerebral bleedings in 18 patients (within 24 h from rt-PA administration in seven, between 1 and 3 days in five, between 4 and 7 days in three, after 7 days in three). According to the criteria of Fiorelli *et al*,^6^ eight patients had haemorrhagic infarctions with small petechial haematoma (HI1), five had haemorrhagic infarctions with more confluent petechiae (HI2), two had parenchymal haematoma less than 30% of the infarcted area, with some mild space-occupying effect (PH1) and three had parenchymal haematoma greater than 30% of the infarcted area with significant space-occupying effect or in a remote area (PH2). The cerebral haemorrhage was symptomatic in two patients (4%).

## DISCUSSION

Stroke accounts for a high proportion of health-resource expenses, which are likely to grow with the population ageing. It is, therefore, important to manage the disease in the most effective way, as European governments are aiming to contain healthcare costs and prioritise cost-effective interventions.^7^

Thrombolytic therapy can change the clinical course of the patient with acute ischaemic stroke, but the 3-h time window requires an efficient organisation and a large number of evenly distributed SU. The treatment delay is due to the late recognition of stroke and to the small number of SU with uneven distribution in the country, so that many patients cannot be transported to specialised centres with experience in thrombolysis within the restricted time window.

A clinical study has already shown that patients with ischaemic stroke can safely be submitted to thrombolytic therapy in the setting of a hospital with experience in the treatment of acute stroke, even without a neuro-intensive care unit.^8^ Patient selection and the following management have to be coordinated and continuously monitored, however, as both are of paramount importance for the best clinical outcome.^9^ ^10^ Our study shows that the selection of patients with acute stroke and their treatment with intravenous rt-PA in the ED is a feasible solution to implement thrombolytic therapy, as the outcome and rate of symptomatic haemorrhages are comparable with NINDS data, provided that: (1) the selection of candidates is based on experienced stroke triage and doctors with experience in stroke management; (2) strict collaboration is established between the ED and stroke team for continuous improvement of stroke care, as recommended by the American Heart Association guidelines;^11^ (3) the patients are submitted to continuous instrumental and clinical monitoring in the following period of high risk of recurrence and neurological deterioration; (4) national and international guidelines are followed with regard to general management and secondary prevention treatment.

This pilot study has some limitations. First, the patients were selected only by stroke team investigators, previously authorised by the Ethical Committee. In the future we plan to instruct all ED doctors in the procedure, so that stroke team alert can be avoided. Second, no patient was treated before 90 minutes. This will be overcome by a larger number of ED doctors with expertise in this treatment, which will reduce symptoms-to-needle time. Third, due to the small number of observations we categorised the outcome in two categories (mRS 0–2 and 3–5), which limits a full comparison with the NINDS study.

Our preliminary experience can be geographically expanded by establishing a network between hospitals. In Germany a collaborative project aims to extend the use of rT-PA treatment to non-urban areas through telemedic transport (TEMPiS).^12^ The telemedic system consists of a digital network that includes a video conference focusing on the clinical examination based on NIHSS and CT/MRI image transfer. Stroke experts may propose the best treatment options and support the administration of intravenous rt-PA, after they have received this information. On the basis of the results of our study, we propose that thrombolysis is performed in the ED of first level outlying hospitals, with the optional support of a stroke team located in third level hospitals (less numerous and more specialised) through televideo consultation. After the procedure, patients can be moved safely to third level referral hospitals to continue specialised treatment. This “drip and ship” approach will offer an extended and effective treatment of stroke, probably at lower costs than if SU were more numerous and spread over a wide territory.
